# A rare case of adenoma of the nonpigmented ciliary epithelium managed with a triple procedure: a case report

**DOI:** 10.3389/fmed.2026.1817040

**Published:** 2026-04-23

**Authors:** Yangyang Jin, Lujia Zhou, Qiuyang Zhang, Xiangzhong Xu, Xiyan Ding

**Affiliations:** 1 The Affiliated Eye Hospital of Nanjing Medical University, Nanjing, China; 2 The Fourth School of Clinical Medicine, Nanjing Medical University, Nanjing, China

**Keywords:** adenoma of the non-pigmented ciliary epithelium, case report, diagnosis, surgical management, triple procedure

## Abstract

**Introduction:**

Adenoma of the non-pigmented ciliary epithelium (ANPCE) is a rare intraocular tumor that is frequently misdiagnosed due to its concealed location and non-specific presentation. This report describes the diagnostic challenges of ANPCE and presents a novel “triple procedure” surgical approach that integrates partial lamellar sclerouvectomy (PLSU) with concurrent phacoemulsification and intraocular lens (IOL) implantation.

**Case presentation:**

A 39-year-old Asian woman presented with a two-month history of progressive visual decline in the left eye, which had been initially misdiagnosed as a cataract at a referral hospital. Slit-lamp examination revealed a mass posterior to the iris at the 10–11 o’clock position. Multimodal imaging, including ultrasound biomicroscopy (UBM) and magnetic resonance imaging (MRI), demonstrated a ciliary body lesion. An incisional biopsy with immunohistochemical analysis confirmed the diagnosis of ANPCE. The patient subsequently underwent localized tumor resection via PLSU combined with phacoemulsification and IOL implantation. Postoperatively, the best-corrected visual acuity (BCVA) improved to 20/22 (0.9), and no recurrence was observed during follow-up.

**Conclusion:**

Unexplained unilateral cataract warrants meticulous slit-lamp and UBM evaluation to exclude occult ciliary body tumors. A staged diagnostic approach may reduce iatrogenic risk, while a triple procedure can achieve complete tumor clearance and restore visual function.

## 1 Introduction

Adenoma of the non-pigmented ciliary epithelium (ANPCE) is an exceedingly rare intraocular neoplasm, accounting for less than 1% of primary intraocular tumors (1). Despite its benign nature, ANPCE poses a diagnostic challenge due to its deep anatomical location and nonspecific clinical presentation. Patients often present with secondary conditions such as cataract or glaucoma, leading to misdiagnosis and delayed intervention (2). In clinical practice, careful slit-lamp biomicroscopy can provide important clues for detecting occult ciliary body tumors. Current diagnostic approaches rely on multimodal imaging, yet ultrasound biomicroscopy (UBM) and magnetic resonance imaging (MRI) often lack the specificity to definitively differentiate ANPCE from malignant ciliary body tumors (3). Furthermore, histopathological examination is regarded as the diagnostic gold standard, although its use may be limited by the invasiveness of intraocular biopsy (4).

In terms of management, surgical resection remains the primary treatment for ANPCE (5). In recent years, increasing attention has been directed toward achieving not only complete tumor removal but also optimal visual rehabilitation (6). Nevertheless, a consensus on standardized strategies for integrated surgical management—such as combined tumor excision, phacoemulsification, and intraocular lens (IOL) implantation—has yet to be reached, particularly in complex cases.

This report describes a rare case of ANPCE in a 39-year-old woman who was initially misdiagnosed with a conventional cataract. We focus on the diagnostic process and the successful application of a “triple procedure” that achieved both complete tumor resection and visual rehabilitation.

## 2 Case presentation

**Figure 1:**
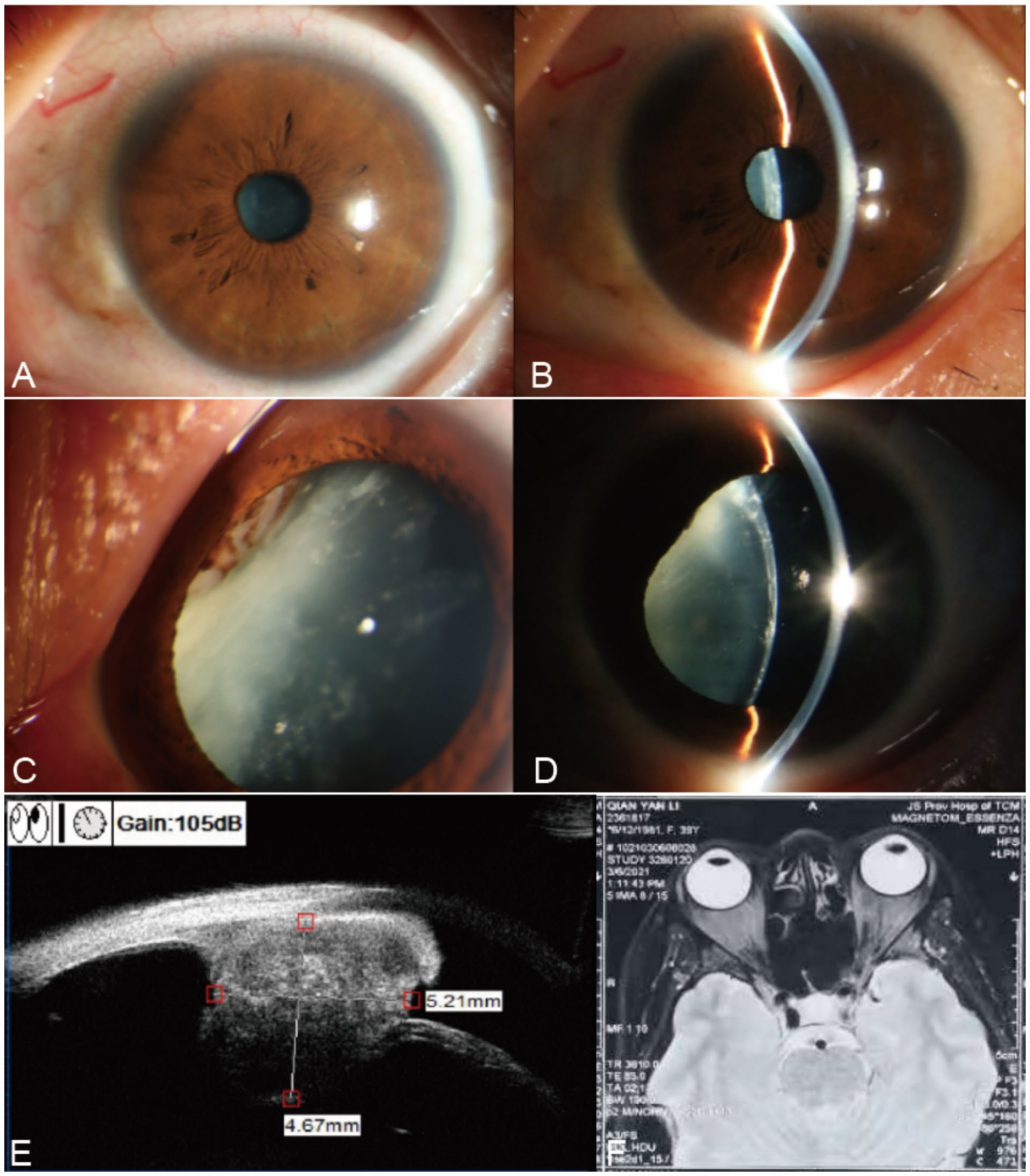
Preoperative evaluation of the left eye. **(A)** Anterior segment photograph showing the overall anterior segment. **(B)** Anterior segment photograph demonstrating anterior chamber depth. **(C)** Anterior segment photograph after mydriasis, revealing a grayish-white ciliary body mass. **(D)** Anterior segment photograph after mydriasis, showing localized lens opacification. **(E)** Ultrasound biomicroscopy (UBM) showing a solid ciliary body mass with medium reflectivity, measuring 5.21 × 4.67 mm. **(F)** Contrast-enhanced MRI demonstrating an enhancing nodule at the 11 o’clock position of the left lens/ciliary body region, indicating a space-occupying lesion.

A 39-year-old woman presented with a two-month history of progressive visual decline in her left eye. She had previously been evaluated at another hospital, where she was tentatively diagnosed with a cataract and advised to undergo surgery. The patient’s medical history was unremarkable for infectious or chronic diseases, and her only prior surgery was a cesarean section performed more than 10 years earlier. On presentation at our institution, uncorrected visual acuity (UCVA) in the left eye was 0.02, and intraocular pressure (IOP) measured by non-contact tonometry (NCT) was 14 mmHg. Slit-lamp examination revealed a transparent cornea, a normal central anterior chamber depth, and a shallow nasal anterior chamber. A localized iris elevation was noted at the 10–11 o’clock position (Figures 1A,B). After pupillary dilation, a gray-white mass with scattered pigmentation was visible posterior to the iris. The mass displaced the iris anteriorly, causing local iris bulging, and exerted pressure on the lens, resulting in localized deformation and opacification (Figures 1C,D). Fundus examination was limited, but the visible retina appeared flat.

Ultrasound biomicroscopy (UBM) revealed a mass with medium internal reflectivity located inferior to the ciliary body at approximately the 11 o’clock position, measuring 5.21 × 4.67 mm (Figure 1E). The provisional diagnosis was a ciliary body mass with secondary cataract. Contrast-enhanced magnetic resonance imaging (MRI) showed an enhancing nodule at the 11 o’clock position of the left lens (Figure 1F), suggestive of a neoplastic lesion, with differential diagnoses including melanoma, ciliary body schwannoma, or hemangioma. Systemic evaluation, including abdominal, breast, uterine, and thyroid ultrasonography, as well as chest and cranial computed tomography, revealed no additional lesions, with no evidence of metastatic disease.

Given the indeterminate nature of the lesion, an initial noninvasive assessment using aqueous humor liquid biopsy was performed. At the time of testing, standardized cytopathology and comprehensive next-generation sequencing (NGS) panels specifically optimized for small-volume aqueous humor samples (<100 μL) in the context of ciliary body lesions were unavailable. Consequently, the aqueous humor analysis was limited to cytokine profiling using the flow cytometric bead array (CBA) method, as summarized in (Table 1). Subsequently, an incisional biopsy of the ciliary body mass was performed under general anesthesia. The mass was accessed through a corneal incision with iris retraction at the 11 o’clock position, and approximately 3 × 3 mm of tissue was excised for histopathological analysis (Supplementary Video 1). A portion of the adjacent vitreous was also collected for cytological examination. Intraoperative findings included inflammatory and tissue cells in the collected vitreous fluid.

**Table 1:** Results of aqueous humor liquid biopsy.

No.	Test item	Result	Unit	Reference range	Method
1	VEGF	10.5	pg/mL	0–40.0	CBA
2	bFGF	4.9 ↑	pg/mL	<1	CBA
3	IL-6	13.0	pg/mL	1.0–50.0	CBA
4	VCAM	245.9	pg/mL	200–1,000	CBA
5	IL-8	9.5	pg/mL	0–20.0	CBA
6	TNF-*α*	12.6 ↑	pg/mL	0–5.0	CBA

VEGF, vascular endothelial growth factor; bFGF, basic fibroblast growth factor; IL-6, interleukin-6; VCAM, vascular cell adhesion molecule; IL-8, interleukin-8; TNF-α, tumor necrosis factor-alpha; CBA, cytometric bead array. The tests were performed by Beijing Zhinde Center for Clinical Laboratory.

**Figure 2:**
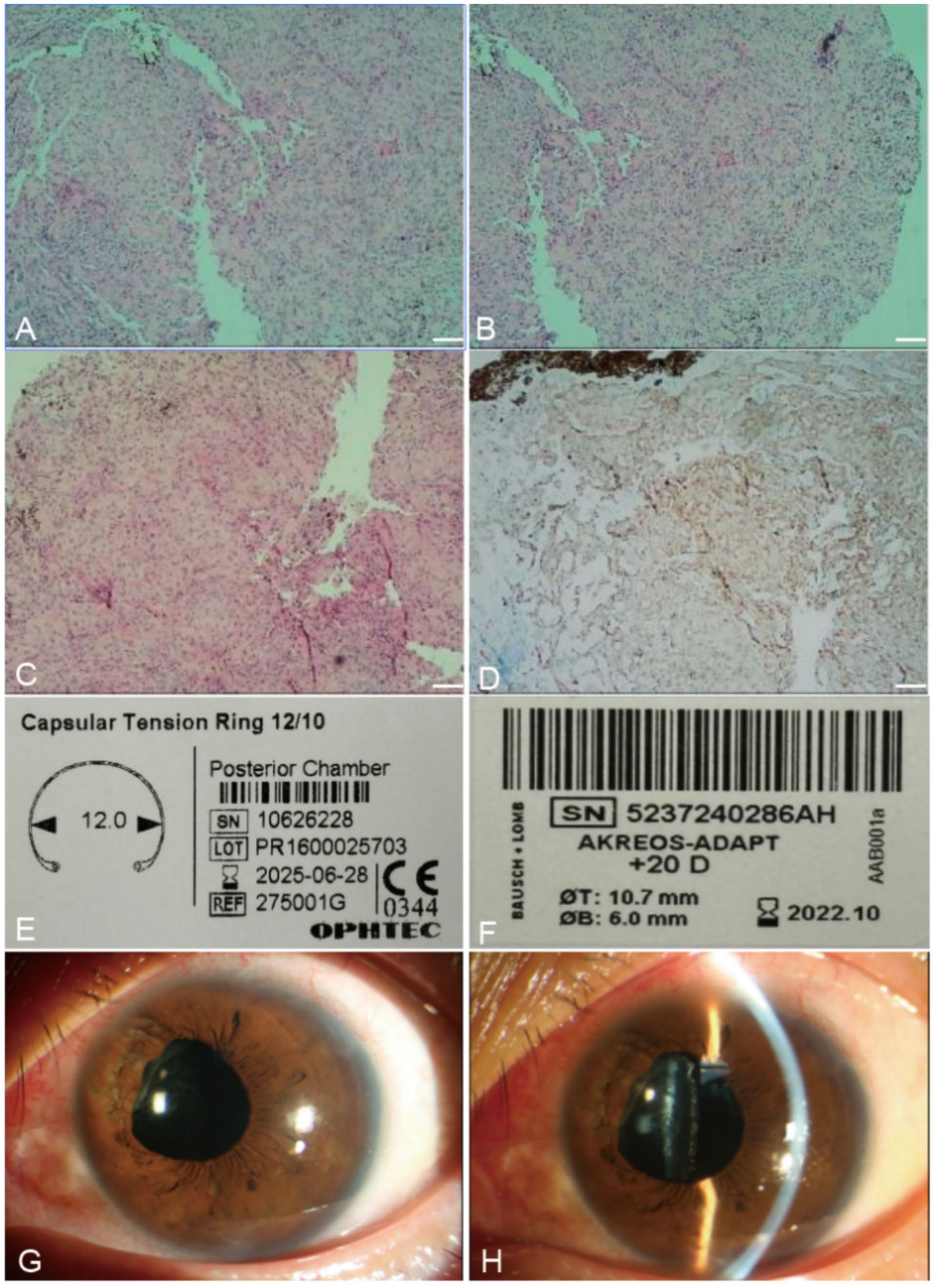
Histopathological findings, detailed parameters of the capsular tension ring (CTR) and intraocular lens (IOL), and postoperative evaluation of the left eye. **(A,B)** Hematoxylin and eosin (H&E) staining showing an epithelial tumor of the ciliary body with cells arranged in nests and trabecular patterns. Scale bar = 100 μm. **(C)** H&E staining reveals tumor cells with moderate cytoplasm and mild nuclear atypia. Scale bar = 100 μm. **(D)** Immunohistochemical staining showing strong cytoplasmic positivity for CKpan. Scale bar = 100 μm. **(E)** Detailed parameters of the CTR. **(F)** Detailed parameters of the IOL. **(G)** Anterior segment photograph on postoperative day 1 showing the overall anterior segment with an irregular pupil. **(H)** Anterior segment photograph on postoperative day 1 demonstrating a well-centered IOL.

Histopathological examination revealed an epithelial tumor consistent with a nonpigmented ciliary origin, composed of cells arranged in nests and trabecular patterns with focal glandular structures (Figures 2A,B). Tumor cells showed moderate cytoplasm, a mildly increased nucleocytoplasmic ratio, and mild nuclear atypia without significant pleomorphism or frequent mitoses (Figure 2C). Immunohistochemical staining was positive for CKpan (Figure 2D) and S-100, negative for HMB45, Melan-A, and SOX10, with a Ki-67 index of approximately 5%. CKpan staining was predominantly cytoplasmic, while S-100 showed both nuclear and cytoplasmic localization. These findings supported the diagnosis of adenoma of the nonpigmented ciliary epithelium (ANPCE).

Two weeks later, to prevent further tumor progression and restore visual function, the patient underwent localized resection of the ciliary body tumor combined with phacoemulsification and intraocular lens (IOL) implantation under general anesthesia. A conjunctival flap was created at 9–11 o’clock, followed by a partial-thickness scleral flap (10 × 4 mm, approximately three quarters of the scleral thickness). The tumor, measuring approximately 8 × 3 mm, was excised along with adjacent vitreous tissue, maintaining a 2 mm safety margin. Minimal intraoperative bleeding (<1 mL) was observed, limited to conjunctival flap creation, scleral flap dissection, and tumor excision. The scleral flap and conjunctiva were sutured. Through a corneal incision, continuous curvilinear capsulorhexis and phacoemulsification were performed. Due to zonular deficiency from tumor compression and resection, a capsular tension ring (CTR) was implanted prior to insertion of a foldable IOL (Supplementary Video 2). Detailed parameters of the CTR and IOL are shown (Figures 2E,F). The surgery was completed without complications, with a total duration of 70 min.

**Figure 3:**
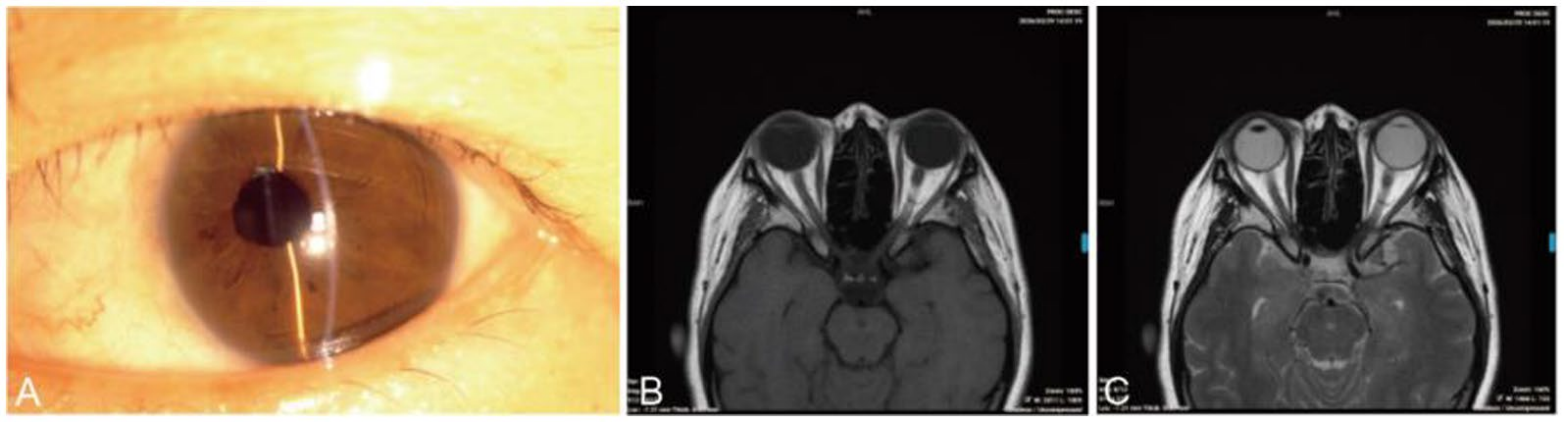
Clinical evaluation of the left eye at 5 years postoperatively. **(A)** Anterior segment photograph at 5 years postoperatively demonstrating a well-centered IOL. **(B)** Axial T1-weighted imaging (T1WI) showing normal signal intensity and morphology of the bilateral globes. **(C)** Axial T2-weighted imaging (T2WI) revealing hyperintensity of the left crystalline lens, with preserved size and normal configuration of the left globe.

On postoperative day one, UCVA in the left eye improved to 0.4 (pinhole 0.6+), and IOP measured by NCT was 23 mmHg. Slit-lamp examination revealed mild corneal edema, a well-positioned IOL, and a shallow anterior chamber with minimal hemorrhage. The fundus view remained limited but appeared normal. The slightly elevated IOP was attributed to minimal postoperative anterior chamber hemorrhage, and no anti-glaucoma eye drops were administered. Postoperatively, the patient received topical levofloxacin eye drops, tobramycin-dexamethasone eye drops (tapered), and tobramycin-dexamethasone ointment for 3 weeks. At 1 month, the best-corrected visual acuity (BCVA) improved to 0.9, IOP measured by NCT was 14 mmHg, the aqueous humor was clear, and the iris at 11 o’clock showed localized adhesion and atrophy with a slightly irregular pupil (Figures 2G,H). The IOL remained well-positioned. At 3-month follow-up, visual acuity remained stable, with no evidence of tumor recurrence. At the 5-year postoperative follow-up, the BCVA of the left eye was 1.0, and the IOP measured by NCT was 18.3 mmHg. Anterior segment photograph showed a well-centered IOL (Figure 3A). MRI demonstrated no evidence of tumor recurrence (Figures 3B,C), indicating sustained postoperative disease control.

## 3 Discussion

### 3.1 Diagnostic challenges of ANPCE

ANPCE is a rare benign neuroepithelial tumor originating from the nonpigmented layer of the ciliary epithelium. Owing to its low incidence and slow growth, clinical manifestations are often nonspecific (5). In this case, the patient was initially misdiagnosed with isolated cataract, consistent with previous reports that ANPCE may cause secondary cataract or glaucoma due to lens displacement, thereby masking the underlying mass (4, 7, 8). This underscores the importance of careful slit-lamp examination and ultrasound biomicroscopy (UBM) in evaluating unexplained unilateral cataract. UBM provides clear visualization of the anterior chamber angle, retroiris space, and ciliary body, enabling accurate assessment of tumor size, margins, and relationship with surrounding structures (3).

Although contrast-enhanced MRI can assist in evaluating tumor vascularity and margins, imaging alone often cannot reliably distinguish ANPCE from ciliary body melanoma or schwannoma. Consequently, histopathological and immunohistochemical analyses—demonstrating CKpan and S-100 positivity coupled with HMB-45 and Melan-A negativity—remain the current diagnostic gold standard (4).

Differentiating ANPCE from ciliary body melanoma poses additional challenges. In adults, malignant melanoma of the ciliary body is more common than epithelial tumors (9–11). In this patient, slit-lamp and intraoperative examination revealed surface pigmentation on the tumor, which differs from the typical appearance of ANPCE reported in the literature (10, 12–14). This pigmentation likely results from displacement or erosion of adjacent pigmented epithelium during tumor growth, as occasionally reported in previous studies (15). Such changes may lead to atypical MRI signals, further complicating preoperative diagnosis. Rare coexistence of iris melanoma and ANPCE has also been documented (14), highlighting the importance of accurate preoperative differentiation for surgical planning. In this case, aqueous humor liquid biopsy failed to detect circulating tumor DNA (ctDNA). Nevertheless, cytokine profiling of the aqueous humor provided additional insights into the tumor microenvironment. Specifically, elevated basic fibroblast growth factor (bFGF) functions as a potent mitogen that promotes tumor cell proliferation and epithelial-to-mesenchymal transition, reflecting the proliferative potential of ANPCE cells (16). Additionally, increased tumor necrosis factor-alpha (TNF-*α*) levels indicate a pro-inflammatory microenvironment that may facilitate blood-aqueous barrier disruption and promote tumor invasion through the induction of matrix metalloproteinases (17). However, these findings were not sufficiently specific for a definitive diagnosis, and imaging features were inconclusive, necessitating tissue biopsy for definitive diagnosis.

### 3.2 Management of ANPCE

The primary goals in managing ANPCE are complete tumor excision, recurrence prevention, and restoration of visual function. Historically, large ciliary body tumors often required enucleation, leading to significant vision loss (18). Advances in microsurgical techniques have enabled local resection to become the preferred approach (5), with partial lamellar sclerouvectomy (PLSU) being the mainstay treatment for ANPCE (6).

Several aspects of this case highlight key surgical considerations and potential advantages:

#### 3.2.1 Staged diagnostic and therapeutic strategy

For cases with an uncertain diagnosis, an initial anterior chamber biopsy—including localized tumor excision and peripheral vitreous sampling—was performed. Compared with extensive primary excision, this staged approach reduces the risk of iatrogenic dissemination in potentially malignant tumors (e.g., melanoma) and facilitates precise planning for subsequent intervention.

#### 3.2.2 Integrated “triple procedure”

Unlike previously reported methods (6, 18, 19), this case combined PLSU-based tumor resection with concurrent phacoemulsification and intraocular lens (IOL) implantation in a single session. This approach simultaneously addresses both tumor removal and cataract-related visual impairment, thereby avoiding secondary surgery and minimizing endothelial and tissue trauma. Complete tumor excision with negative margins was achieved, scleral integrity was preserved, and adjunctive vitreous management minimized the risk of retinal detachment and macular edema. In this case, the “triple procedure” proved to be feasible for a ciliary body tumor associated with secondary cataract, particularly when the lesion involved a limited portion of the zonules (<3 clock hours) requiring partial zonular excision, and when intraocular pressure remained stable without advanced secondary glaucoma.

#### 3.2.3 Application of a capsular tension ring

Tumor location in the ciliary body necessitates partial sacrifice of the zonular fibers. Traditionally, this has precluded primary IOL implantation or required secondary scleral fixation (9). Implantation of a CTR supports the capsular bag, maintains IOL centration, and reduces anterior vitreous prolapse (20). In the present case, these advantages contributed to the patient’s favorable postoperative visual outcome (BCVA 0.9). Key considerations for CTR placement include implanting the ring after thorough cortical aspiration but before IOL insertion (21), thereby allowing full expansion within the capsular bag without trapping residual cortical material. The leading end of the ring should be directed toward the area of zonular deficiency to redistribute stress and protect the remaining intact zonules (22).

Despite the satisfactory clinical outcome, several limitations remain. Preoperative aqueous humor cytokine analysis [e.g., VEGF (23), bFGF, TNF-*α*] indicated alterations in the intraocular microenvironment but lacked specificity. Incorporating next-generation sequencing (NGS) to detect tumor-specific circulating tumor DNA (ctDNA) or microRNA (miRNA) profiles could enable noninvasive molecular diagnosis, particularly for distinguishing ANPCE from malignant melanoma (24, 25). Moreover, the use of intraoperative OCT (iOCT) could be optimized to provide real-time visualization of tumor margins and the integrity of the ciliary body during resection, potentially reducing iatrogenic trauma to the iris and pupil (26). Additionally, long-term tumor compression and surgical manipulation may lead to postoperative pupil deformities, emphasizing the need to balance complete tumor excision with preservation of iris structural integrity.

## Data Availability

The raw data supporting the conclusions of this article will be made available by the authors, without undue reservation.
