# Supplementary material for: A rare case of adenoma of the nonpigmented ciliary epithelium managed with a triple procedure: a case report
Source: Front Med (Lausanne). 2026 Apr 23;13:1817040. doi: 10.3389/fmed.2026.1817040 (PMC13149164; doi:10.3389/fmed.2026.1817040)
Supplement: Supplementary file 1 [file Table_1.docx]

Supplementary Video 1. Incisional Biopsy of a Ciliary Body Mass

Core content: This video demonstrates incisional biopsy of a ciliary body mass in the left eye under general anesthesia. A clear corneal incision was made at 11 o’clock, and the lesion posterior to the iris was exposed using iris hooks. A tumor specimen about 0.3 cm in diameter was excised with intraocular scissors for histopathology. Limited anterior vitrectomy was performed to obtain aqueous and vitreous samples for cytology. Total surgical time was 50 minutes. Minimal bleeding occurred during corneal incision and tumor excision.

Duration: 4:34

Shooting angle: Coaxial view under an operating microscope.

Editing notes: Edited from the full surgical recording due to file size limits. Preoperative preparation and postoperative suturing were omitted, while all critical intraoperative procedures were preserved.

Supplementary Video 2. Tumor Resection Combined with Phacoemulsification and IOL Implantation

Core content: This video shows definitive tumor resection following biopsy. A partial-thickness scleral flap (9–11 o’clock) was created, followed by complete tumor excision. Phacoemulsification was performed via a separate incision. Due to zonular instability, a capsular tension ring (CTR) was implanted prior to foldable intraocular lens (IOL) insertion. Minimal bleeding occurred during the creation of the conjunctival and scleral flaps, as well as during tumor excision.

Duration: 3:58

Shooting angle: Coaxial view under an operating microscope

Editing notes: To comply with file size restrictions, the video was professionally edited and compressed. Time-consuming steps (e.g., scleral flap dissection, viscoelastic injection, and irrigation/aspiration) were accelerated or condensed, while all critical surgical steps and procedural continuity were preserved.
